# Simulating real-life scenarios to better understand the spread of diseases under different contexts

**DOI:** 10.1038/s41598-024-52903-w

**Published:** 2024-02-01

**Authors:** Rafael Blanco, Gustavo Patow, Nuria Pelechano

**Affiliations:** 1 ViRVIG, Universitat Politecnica de Catalunya, 08034 Barcelona, Spain; 2 ViRVIG, Universitat de Girona, 17003 Girona, Spain

**Keywords:** Computer science, Applied mathematics

## Abstract

Current statistical models to simulate pandemics miss the most relevant information about the close atomic interactions between individuals which is the key aspect of virus spread. Thus, they lack a proper visualization of such interactions and their impact on virus spread. In the field of computer graphics, and more specifically in computer animation, there have been many crowd simulation models to populate virtual environments. However, the focus has typically been to simulate reasonable paths between random or semi-random locations in a map, without any possibility of analyzing specific individual behavior. We propose a crowd simulation framework to accurately simulate the interactions in a city environment at the individual level, with the purpose of recording and analyzing the spread of human diseases. By simulating the whereabouts of agents throughout the day by mimicking the actual activities of a population in their daily routines, we can accurately predict the location and duration of interactions between individuals, thus having a model that can reproduce the spread of the virus due to human-to-human contact. Our results show the potential of our framework to closely simulate the virus spread based on real agent-to-agent contacts. We believe that this could become a powerful tool for policymakers to make informed decisions in future pandemics and to better communicate the impact of such decisions to the general public.

## Introduction

During the COVID-19 pandemic, there were many attempts to closely simulate the spread of the virus^1–3^ and the impact of wearing masks^4,5^, respecting social distancing^6^, and forcing total or partial lockdowns in cities^7^. Many of these decisions were based on statistical models^1,2,8^ that tried to simulate the probability of the virus spread based on such decisions. Having an accurate virus spread model^9–14^ requires two key elements: (1) fully understanding the virus and how it spreads from person to person, and (2) realistically simulating the interactions between people. The first one was extensively used and updated as new information about the virus’ behavior was discovered by doctors and epidemiologists^3^. However, the second one was not developed in such depth due to the complexity of creating detailed, controllable crowd simulation models that can accurately replicate such behaviors.

Having an accurate crowd simulation model requires modeling agents that can exhibit behaviors close to real people. More specifically, agents should have a home assigned in the environment, go to a specific job every weekday, shop in nearby supermarkets, and sometimes eat out at restaurants. If we can simulate real everyday behavior for many agents, then we can have a more accurate simulation of the virus spread, since we know which agents live in the same house or interact with each other at work. We could also simulate the differences between what happens if the waiter at a restaurant is infected as opposed to a client who spends little time at the restaurant.

In this work, we set out to build a framework of autonomous agents with detailed interactions among themselves (i.e., agent-agent) and with the environment (i.e., agent-environment). These interactions can be traced now up to an unprecedented level of detail, allowing a very detailed analysis of contagion situations. We enhance our system with a further layer of contact tracing taking into account protection factors such as wearing masks and other restrictions, which would enable stakeholders to make difficult decisions much like in the recent Covid-19 pandemic. Since modeling the virus itself is out of the scope of this work, in this paper, we will use a general parameterized virus model that could be adjusted to simulate different viruses. What we are interested in is being able to replicate human interactions to obtain an accurate simulation of virus spread based on contact between infected and healthy individuals. In addition, one of the biggest problems during the last pandemic was the difficulty for the general public to understand the decisions made by policymakers, e.g., the need for the correct wearing of masks. Since the public often had the feeling that these decisions were random, it was difficult to force them to respect the constantly changing rules. Our tool could help simulate and visualize the impact of these decisions so that the general public can better understand why certain restrictions should be imposed.

The main contributions of this work are (1) a modular framework to simulate human whereabouts and interactions (agent-agent and agent-environment) consistently, enabling the simulation of contagion models more accurately; and (2) a visual tool for policymakers to better communicate the impact of their decisions. Our framework can handle the traceability of people to simulate virus propagation based on the duration of exposure to other infected people under different conditions (e.g., distancing or mask usage). In our framework, an agent could be followed through the day or week and its behavior would resemble a real person (e.g., following a timetable, similar routes every day between home and work, and attendance to nearby services based on needs such as doing groceries or eating out). Routines are automatically adjusted based on the state of the world, and new building types or objects could be added with their corresponding interaction details so that the agents would be able to automatically interact with them.

### Virus spread models

In recent times, public health decision-makers have relied more and more on mathematical models that can project how infectious diseases progress to show the likely outcome of an epidemic. In general, mathematical models start from a set of basic assumptions, or collected statistics, to find parameters for various infectious diseases, using those parameters to calculate the effects of different interventions, such as population restrictions (e.g., mobility) or mass vaccination programs. The use of these modeling tools can help decide which interventions to avoid and which to try or can predict future growth patterns. In recent years, the literature on these models blossomed due to the COVID-19 pandemic, resulting in hundreds, if not thousands, of new publications each year. One of the most popular approaches is compartmental models, which are a very general modeling technique. In these models, the population is assigned to compartments with labels—for example, S, I, or R, (Susceptible, Infectious, or Recovered)^15^, called the SIR model. People may progress between compartments. The order of the labels usually shows the flow patterns between the compartments; for example, SEIS means susceptible, exposed, infectious, then susceptible again, being one of the most popular models to describe the Covid-19 epidemic. The resulting model can be then solved either by using direct deterministic differential equation solvers^8^, which is by far the most popular and widely used approach; or using the well-known stochastic Doob-Guillespie algorithm^16^, which was originally developed in the context of chemical reactions but that also has been successfully applied to disease propagation^17^. For a comprehensive review of modern developments in the area, we recommend the interested reader refer to the book by Kuhl^3^.

#### Spread simulation frameworks

However, efforts for modeling these mathematical models in the context of actual populations, considering all their nuances and particularities, are complex and difficult to implement. One of the most relevant works is the one by Silva et al.^18^, who develop a multi-level framework for a simulation of the coupled effects of environment and population, based on the BioClouds framework^19^. BioClouds, in turn, is based on the key idea of simulating aggregation of agents as singular units (a *cloud*), providing more accurate simulations taking into account the agent’s (i.e., both individuals and clouds) velocities and densities. To simulate the contagion process, Silva and coauthors used the SIR model mentioned above. However, this system does not exhibit logical whereabouts for individuals, so if we trace a single agent we may notice that it goes to a different home each day of the week, and thus we cannot trace the contagion pattern of individuals.

Examples of remarkably popular simulation environments are Emulsion, GAMA, NetLogo, and Repast. Emulsion^9^ is a generic simulation framework, originally developed in the context of animal epidemiology but then extended to humans and their complex interactions, based on multi-level multi-agent modeling. It uses a Domain-Specific Language to let the user define the different components of an epidemiological model, such as assumptions, model structure, or model parameters. In spite of all this flexibility, its implementation is not tailored to controlling the details of the interactions and modeling agent behaviors in a realistic way, as our framework does. On the other hand, GAMA^10^ is an open-source modeling and simulation environment for creating spatially explicit agent-based simulations with the flexibility of domain in mind. GAMA has a high degree of openness, which allows the addition of plugins for specific needs, as well as creating multi-level simulations, for instance combining an agent-based simulation for open spaces while using differential equations for building interiors. This framework shares many features with our proposal, but our framework allows a fully individual-based tracking, including not only agent-agent interactions, even at the casual, street-level but also agent-environment interactions through a programmatic interface, thus providing a general simulation environment. NetLogo^11,12^ is a multi-agent programmable modeling environment, mostly intended for disease spread studies in "open" environments, although a GIS-importing module has been added. It has general programming capabilities, which provide a great deal of flexibility, but not at the level proposed in our framework. Repast^13,14^ is a set of software tools for agent-based modeling and simulation. The core concept in Recast is the *events*, which are driven by a discrete-event scheduler in the simulations, associated with concrete time points (ticks). Interactions between agents are controlled, through code snippets, at these discrete time points. Instead, our framework works on a flexible time representation that allows the combination of continuous agent movement with discrete-time interactions. Simulation libraries and platforms dedicated to epidemiological issues are rising, e.g. SimInf^20^, an *R* library for data-driven compartment-based models; MicroSim^21^, an agent-based platform for several kinds of diseases; or GLEaMviz^22^, a population-oriented platform for simulations at the global level. Finally, Broadwick^23^, is a Java framework that uses Approximate Bayesian Computation (ABC) and Markov Chain Monte Carlo (MCMC) methods for both compartment- and individual-based models. Broadwick also uses interaction networks.

We want to emphasize that, in all these cases, these approaches still require writing large portions of code to derive specific classes and carry out simulations on practical situations, while lacking detailed interaction handling capabilities that are not provided as platform features, which may be added with deep coding and great efforts. Although some of these software packages are quite sophisticated, they do not deal with agent-agent or agent-environment interactions with the level of detailed control that our framework provides.

### Crowd simulation models

A virtual city is mainly composed of two elements: the buildings and objects that compose the geometry of the city; and the virtual humanoids that inhabit it. Previous work in this area has presented different ways for creating and authoring the integration of these two components, achieving humanoids that move from one side to another with a certain purpose or activity to pursue.

#### Authoring trajectories

Early work by Yersin et al.^24^ used a semantically augmented navigation graph for defining the different zones of interest and directing, through a GUI, the crowd movement. Recently work from Mathew et al.^25^ proposed another authoring tool that uses a sketching language to convert sketching gestures into crowd simulations, where the language allows to refine parameters such as path, thickness, density, and velocity. In another work, Jorgensen and Lamarche^26^ worked with agents that construct schedules and agendas with the most appropriate route for performing a set of tasks before reaching an end-point, under spatial, temporal, and personal characteristics constraints.

#### Framework and approaches to populate an environment

CAROSA framework presented by Allbeck^27^ uses Microsoft Outlook’s calendars format as a scheduling interface for task definition, being able to specify the groups or individuals, time, and location related to each activity. This framework was developed considering non-expert users like artists. In another work, De Paiva et al.^28^ presented the UEM (Urban Environment Model) that consists of a type of simulation where there are roles with predefined schedules that are assigned to the agents depending on semantics like age. These roles guide the movements and stays of the agents during the simulation. Similarly, Li and Albeck^29^ define roles through activities that indicate the place and the way agents should behave according to the simulation time. Furthermore, they implement a role-switching functionality that allows agents to behave differently according to the simulation conditions, like the location, needs, and reactions to other agents.

#### Actions and descriptions in natural language

Badler et al.^30^ defined a model called Parameterized Action Representation (PAR) which was used to translate actions written in natural language into instructions that can be understood and executed by the agents of his system. In addition, he suggests the creation of a structure called *Actionary* which is a set of PARs with well-known and refined actions. We use the same concept of the PARs to define the interactions in the places of our simulation, with the difference that the format is different and these definitions are not grouped in a general dictionary, but each object keeps its parameters to be used. Similarly, the work of Mainardi et al.^31^ allows the creation of a simulation with agents, groups, and crowds, being able to define behaviors that depend on time or location using an interpreter that translates a set of actions written in a scripting language very similar to natural language.

#### Environments semantically augmented, smart objects

The work of Kallmann and Thalmann^32^ demonstrates the potential of defining within the same object all the necessary information needed to interact with it, especially in terms of reusability and decentralization. Another approach in the context of environmental planning by Tabak et al.^33^ consisted of the study of human movement in indoor office-building spaces and normal working conditions, comparing the results with empirical data obtained by web-based diaries and radio frequency identification technology. Simulations were defined based on basic standardized activities (called skeleton activities) with interruptions from "intermediate" activities, always defined within the context of the office environment defined. Similarly, Simeone and Kalay^34^ proposed the use of AI engines, in particular Finite-State machines, to control agent behavior within a working environment. Schaumann et al.^35^ defined narratives (a formalized set of instructions the agent should follow) with possible adaptations to changing conditions in the context of hospital settings, to assess daily movement patterns. Recently, Rogla et al.^36^ presented a framework for generating a populated environment using procedural techniques. They employ rule-based grammars to generate agendas for each humanoid; this technique offers the possibility of reusing, modifying, and extending previously generated populations by editing the behaviors’ file, but does not provide the flexibility of the approach presented here, where detailed activities can be defined and used to track interactions among agents. The already commented work by Silva et al.^18^ presented LODUS, a framework for the simulation of virtual urban environments with various levels of detail, based on their BioClouds^37^ model to run the simulation at the macroscopic level, creating groups of individuals with the same characteristics to move between the different points of interest of the city; then, they perform experiments in a microscopic level of detail for considering other scenarios where the phenomenon studied required a closed view of the crowd interactions like the social distancing and its impact in the COVID-19 contagious process.

The interested reader is referred to the recent survey by Lemonari et al.^38^ for an in-depth review of the state-of-the-art literature on authoring crowd simulation.

#### Crowds and epidemics

Usman et al.^39^ evaluated navigational policies within closed environments (e.g., a shopping building) to measure a social safety distancing index. Their approach used pre-defined paths (the navigational policies), tracking possible interactions between subjects when their social spaces (a bounding volume) intersect. Comai et al.^7^ studied the re-opening steps as required by Italian protocols and regulations as a preliminary measure for the re-opening of an educational building. They acquired three-dimensional geometry with laser scans, and used crowd simulation software to populate the environment, measuring social distancing. Harweg et al.^40^ proposed an agent-based pedestrian simulation to assess their interactions in public places for contact tracing for infectious diseases like COVID-19, gathering insights about the effectiveness of distancing measures. In their implementation, a force-based system was used to move individuals, and interactions were accounted for when the distance between agent centers was below a given threshold. Wang et al.^6^ used indoor simulations of randomly moving agents to illustrate COVID-19 and distancing measures for the general public. Rahn et al.^41^ used randomized destinations for the agents within a building to analyze their interactions in combination with a virus (COVID-19) spread model to qualitatively assess the risk of exposure. Lv et al.^42^ embedded an infection model into a crowd simulator, also to assess COVID-19 transmission on a university campus. Each agent performed a closed-loop trajectory (dorm-class-dorm) using the Dijkstra shortest path algorithm for the trajectories. Interactions were also computed by measuring agent bounding geometry collisions. Comai et al^43^ developed a methodology to reorganize spaces in school buildings to allow safe reopening following the COVID-19 pandemic. For this, they developed specific situations such as school entry/exit and lunch break, coded through deterministic rules coded into the Unity game engine. The resulting applications were used by stakeholders to make decisions but also to educate children about the correct behaviors in these pandemic times.

## Methods: semantically linked city-crowd framework

We created a simulation framework constituted of a city, agents, and agendas. These agendas are built using the semantics of the city and serve as a link between the two main components, i.e., the city and the crowd. The city contains roads and buildings with apartments, locals, and offices. The agents can navigate the city in the exteriors and interact with the places in their interiors. Our simulator was built with the intention of considering those interactions between the city elements and the virtual agents in a way that cannot be handled by probabilistic models of virus spread. For example, our system can simulate close interactions between agents that share a place for a period of time and how this could lead to contagion. Furthermore, our simulator tries to replicate in detail the movement of the agents inside the city and a set of the activities that they perform every day. The system allows us to observe coherent behaviors, movements, and scenarios; such as agents that are assigned to a household will leave from it at the beginning of their day and come back to it when finishing with all the activities allocated for the day. Likewise, the offices and desks are assigned to the agents and will be *persistently* maintained throughout the simulation.

### Generating semantically augmented cities

The simulator is composed of two main elements: the semantically augmented places and the agents. They were designed and programmed using the Unity 3D game engine^44^, which allowed the use and merge of prefabricated models to build city buildings and agents. In addition, its navigation mesh was used so that the agents could find their way between the different objectives they had assigned on a daily basis. These objectives are provided by their individual agendas, which serve as the link between the city semantics and the agents. The semantics of the elements in the city and the behaviors are expressed by the user. One of the novelties of our semantically augmented model is that part of the semantics of a place includes the algorithms that indicate how the agents must behave inside it. It means that the movements, rotations, animations, and waiting times are organized and stored as the behaviors of the agents that want to interact with the place. The places developed for the experiments are apartments (i.e., households), offices, restaurants, and supermarkets. There are office buildings that contain a set of desks and chairs for agents to sit and work. Restaurants are establishments filled with tables and chairs, a kitchen, and a welcome desk where a set of agents who work in the restaurant, and other agents who will be served, participate and interact. Moreover, supermarkets are spaces with a set of shelves and a cash register where agents walk around in search of the items they wish to purchase. Each of the objects contained in these locations has instructions that the agents consult when interacting with them. Our framework follows a modular design to facilitate re-usability and extensions of existing models with semantics. So for example, when the user wishes to include a new model which should behave in a similar manner to an existing one, it can simply copy the semantics into the new object. This is, for example, the case of the queue formed at the welcome desk of a restaurant and at the cash register of a supermarket. Building a new city or extending an existing one, can be easily achieved by simply replicating these smart objects and/or entire places/buildings/blocks. The virtual agents will then be able to effortlessly interact with them. All the elements in the system can be replicated to further extend or refine their semantics and interaction instructions, which makes our system flexible and extendable.

The second key element of our model, the agents in the crowd, relate to the city and its semantics through agendas that describe their movements around the day. These agendas are generally a list of interaction tasks with places that indicate the hours at which the agent should be moving toward them and the length of time needed to perform each task. The behaviors inside the places are stored within the places themselves and retrieved when the agents need them. Therefore, after an agent arrives at a location and interacts with it, it receives a list of instructions describing movements, waiting, activation of animations, and interactions with other objects within the scope of the location, which will provide another list of instructions to be performed. Agents keep track of their daily activities to guarantee consistency in their behaviors. These agendas are generated automatically at the beginning of the simulation based on the agent’s role within the crowd, and the places available in the city. So we guarantee consistency of the agent’s behaviors throughout the day.

As an illustration of our places that store behaviors, let us take as an example the interactions performed inside a restaurant in our model. When the agent arrives, the first step would be to interact with the welcome desk, which will also have its own behaviors to follow, such as positioning correctly in the queue to wait to be attended to. Next, the agent will move to the table assigned, interact by sitting in a chair, and wait several seconds to call the waiter; then, he will order, wait for the food, eat when the food arrives, and walk to the cash register to pay for the food. This sequence of steps is stored in the restaurant component, and it is also subdivided into independent components; for example, the welcome desk and the cash register are independent components used in the interaction that can be replicated in other situations, like the cash register of a supermarket. Note how our system allows us to accurately follow a simulated agent through the day and keep track of visited locations and the length of time that it is been in close contact with other agents. Also, since we follow consistent agendas driven by the behavior of the places, our agents will consistently meet with the same virtual agents in the office every day, and likely a small group of agents that live in their neighborhood and thus are likely to choose the same restaurants or groceries.

### Crowd simulation

**Figure 1:**
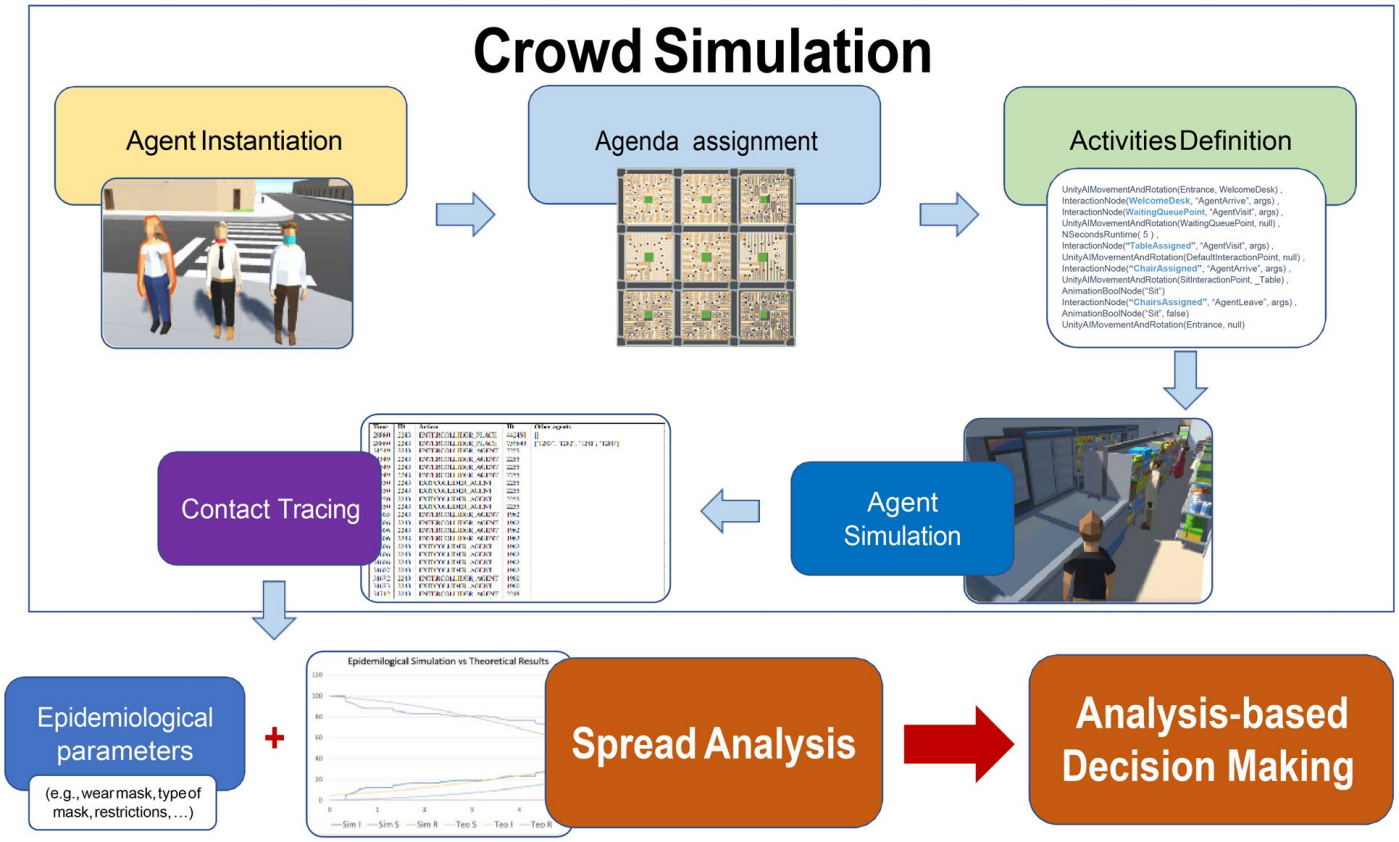
Overview of the proposed system. On the top, is the first stage where agents are simulated. First, agents are instantiated and activities are defined, e.g., family, work, grocery buying, etc. This information is recorded, tracing individual contacts and individual interactions. Then, this is re-used in a second stage for virus spread assessment, taking into account different epidemiological parameters such as wearing/not wearing masks, type of masks, and social restrictions. Finally, with all the gathered information and analysis, informed decision-making is possible.

As can be seen in Fig. 1, our system works in two stages: first, crowd simulation, where crucial information about the agents’ whereabouts and interactions is recorded. Then, in a second, independent stage, this information is used, together with epidemiological parameters (e.g., mask usage, type of mask, restrictions, etc.) to assess the spread of the virus among the individuals that were traced in the first stage. This enables the simulation of multiple scenarios in a fraction of the time that would require a full-blown simulation each time.

#### Agent instantiation and agenda assignment

The simulation starts by processing the number of apartments added to the map and instantiating an agent in each available space within the apartments. There are 2- and 4-people apartments. Each time an agent is instantiated, it automatically gets an agenda assigned, which it will have to fulfill until the end of the simulation. There are different types of agendas for the different types of workers in the simulation, such as supermarket, office, or restaurant workers. These agendas are converted into a list of interactions to be performed according to the simulation time. As mentioned, agent agendas are randomly initialized with different tasks for each agent, filling its daily activities. These activities include different tasks for working days and weekends and remain unchanged during the simulation time. However, if the simulation is rerun, as agendas are randomly assigned, then agents will behave differently from the previous simulation. Although, in our current implementation, agendas for a given agent have the same activities for working days (but different for different agents), observe that there is no reason an agent could not have different activities for each day, as it is simply a matter of initializing with different structures for each day, the same way we have different activities for working days and weekends.

#### Activities definition

There is a main clock that will guide the agents as to what action they should be taking at any given moment. Agendas consist of a list of actions that describe an interaction with a place for a period of time. There are actions that are not bounded by time and are performed just after the end of the previous action, for example, trips to the supermarket. There are other actions that have a start and end time, such as work activities. Any interaction can be simulated as a small black box; i.e., without internal structure; or can be decomposed into smaller activity snippets, which could be generated automatically with known algorithmic tools^45^.

#### Time representation

Our simulation uses a continuous/discrete time model based on Unity’s own game update system. All agents are updated with Unity’s *Update()* method, which calls each agent, in turn, to represent an instant in the simulation, although nothing prevents parallel execution of the agents on a multi-processor machine. Among other activities, Unity updates agent animations, for visualization purposes only; detects collisions, which are used for agent-agent interactions; and moves forward the main clock mentioned before, triggering agent- and agenda-based events. Then, when an agenda-based event is triggered (e.g., an agent needs to leave work to go and pick children up from school), the agent switches its current activity for the new one and continues behavior as usual.

#### Agent-agent and agent-environment interactions

Given that our agents have a visual representation, we use capsule colliders not only to detect collisions with other agents but also for obstacle avoidance, as usually done in crowd simulation systems^27,31,36^. Whenever a collision between two agents is detected, a local timer is activated and, if the collision takes longer than a user-defined threshold, an interaction is recorded for further treatment, see below.

On the other hand, in our system, every agent can interact with its environment not only by colliding with its geometrical elements but also by interacting with objects following a set of instructions provided by the object itself. An example of these detailed interactions is presented below, in “Case studies” section and Fig. 5, where the interactions between agents and a supermarket are described. These interactions are encoded, not in fixed snippets of code within the agents, but are associated with the environment itself (i.e., with the supermarket), being easily configurable by changing the interaction description. Thus, every interaction is decomposed into atomic steps (e.g., go to the shelf, take a product, iterate these first two steps until satisfied, go to the cashier, pay), associated with each location. Thus, agents do not need to know how to interact with any store (or any other environment location), but simply retrieve the operational instructions and follow them. This, of course, will produce more interactions with other agents also interacting simultaneously with the same environment. See the description below.

#### Recorded information

As the simulation runs, important information for an epidemiology study is collected. The first information collected is the close contact between agents. Close contact happens when the agents are close to each other during a specific length of time, therefore we also record information regarding the time when two agents start a close contact and the time when the contact finishes. Formally speaking, a close contact, *C* (*A*_*i*_*, A*_*j*_) between agent *A*_*i*_ and *A*_*j*_ happens when the distance between the agents is below a user-defined threshold *δ*, *distance*(*A*_*i*_*, A*_*j*_) < *δ*, during a length of time larger than *τ*_*c*_ seconds. In our simulation *δ* = 1*.*5 m and *τ*_*c*_ = 5 s.

Secondly, data is collected on the places where the agents are located; the time when they arrive and leave closed places is recorded. We thus keep track of the viral load of an enclosed space due to infected agents visiting it. The viral load *V*_*L*_ represents how infectious is the air in an enclosed site, and it will increase based on the number of people infected and the length of time they stay within the site. Therefore:

1 $$V_{L} = \gamma \sum\limits_{n = 1}^{{N_{A} }} {A_{n} }$$

where *N*_*A*_ is the number of agents in the site, *A*_*n*_ is a binary number representing whether the agent is infected (*A*_*n*_ = 1) or not (*A*_*n*_ = 0), and *γ* represents the probability of disease transmission (i.e., contagion) for each person in a risk situation, at every simulation step. This value can be defined by the user and thus adjusted to the virus properties. In our simulation, we used *γ* = 0*.*05.

Finally, the infection process considers also the percentage *p* of protection offered by the mask worn by the agent, to lessen the degree of contagion when masks are being used by the agents. The probability of being infected when wearing a mask *M*_*p*_ is calculated as:

2 $$M_{p} = 1 - \frac{p}{100}$$

where *p* is the protection percentage of the mask divided by 100. So for example a mask offering 95% protection will have *M*_*p*_ = 0*.*05.

If we incorporate the mask protection percentage into Eq. (1), we have:

3 $$V_{L} = \gamma \sum\limits_{n = 1}^{{N_{A} }} {A_{n} M_{p} \left( {A_{n} } \right)}$$

This simulation described through records is processed to study how the virus spreads among the agents of the original simulation, the main methods of contagion the close contact between agents, as well as the permanence of an agent in a closed place where there are other infected agents.

### Disease simulation

With the results of the crowd simulation, we used the framework developed to run Doob-Guillespie-like disease propagation simulations. By varying the parameters of our model, we can compare results regarding the impact or effectiveness that the different measures taken against COVID-19 had in the spread of the virus. In our model, we contemplated whether citizens were wearing masks or not, or wearing them in the wrong way (e.g. mask below the nose or the chin) just like we could observe in the real world.

The simulation is initiated considering a percentage of the entire population infected, which is 5% for our experiments but it can be varied as needed. There are two ways in which contagion can occur: by direct contact with an infected agent for more than a certain period (5 s for our experiments), or by sharing a closed place with an infected agent. It should be noted that these scenarios initiate a contagion process that will depend on the type of mask used by the agents involved. We also consider the use of different types of masks whose effectiveness against the virus varies between 75 and 98%; this number will thus indicate the agent protection level when wearing such mask, called *protection factor (PF)*^46^.

It means that when a contagion process is initiated caused by enduring contact or the sharing of a place with other infected agents if the PF is below a uniform random number, the agent will be infected.

To take into account that some people are not easily infected, we assume that agents not wearing masks have a PF against the virus of 10% (*M*_*p*_ = 0.9). Finally, any agent wearing a mask in the wrong way will have the PF of the mask reduced by 50%^46^. This parameter can be adjusted as needed with the aim of accurately simulating the most realistic scenario.

Therefore, when an agent *A*_*i*_ is in close contact with an infected agent *A*_*j*_, we compute the contagion as follows: We first compute a random value between 0 and 99 which will drive the contagion process:

$$\rho = random({1}00)$$

and then the contagion will depend on *ρ* and the combination of mask protections from both *A*_*i*_ and *A*_*j*_:

4 $$A_{i} = \left\{ {\begin{array}{ll} {1,} & \quad {{\text{if}}\;\rho \le M_{p} \left( {A_{i} } \right) \cdot M_{p} \left( {A_{j} } \right) \cdot 100} \\ {0,} & \quad {{\text{otherwise}}} \\ \end{array} } \right.$$

The other case for an agent to get infected is due to being in a site with infected agents for a period of time longer than *τ*_*s*_ (in our simulation we use *tau*_*s*_ = 60 min). So given a random value from 0 to 99:

$$\rho = random({1}00)$$

An agent *A*_*i*_ infection process due to being in a site for more than *τ*_*s*_ seconds is:

5 $$A_{i} = \left\{ {\begin{array}{ll} {1,} & \quad {{\text{if}}\;\rho \le M_{p} \left( {A_{i} } \right) \cdot V_{L} \cdot 100} \\ {0,} & \quad {{\text{otherwise}}} \\ \end{array} } \right.$$

To simplify the model, we have assumed that the mask protection affects equally how it protects the agent wearing it and those in close contact with the agent. However, we could easily extend the model to consider two different types of protection per mask based on whether it is self-protection or protecting others.

### Visualization

Unity game engine was used to create the city and the agents that inhabit it. A set of prefabricated objects was used to model the buildings and other components of the city. In the execution of the simulation, we can observe how the agents walk and move between the different points of interest, making contact with other agents. We can also observe how they interact with objects such as chairs, tables, and desks. we avoid using abstract representations of objects and work with figures that look like humans and structures and objects that look like those of a real city as we believe that this can help stakeholders to better visualize the scenarios, allowing them to identify problems and possible measures to solve them.

**Figure 2:**
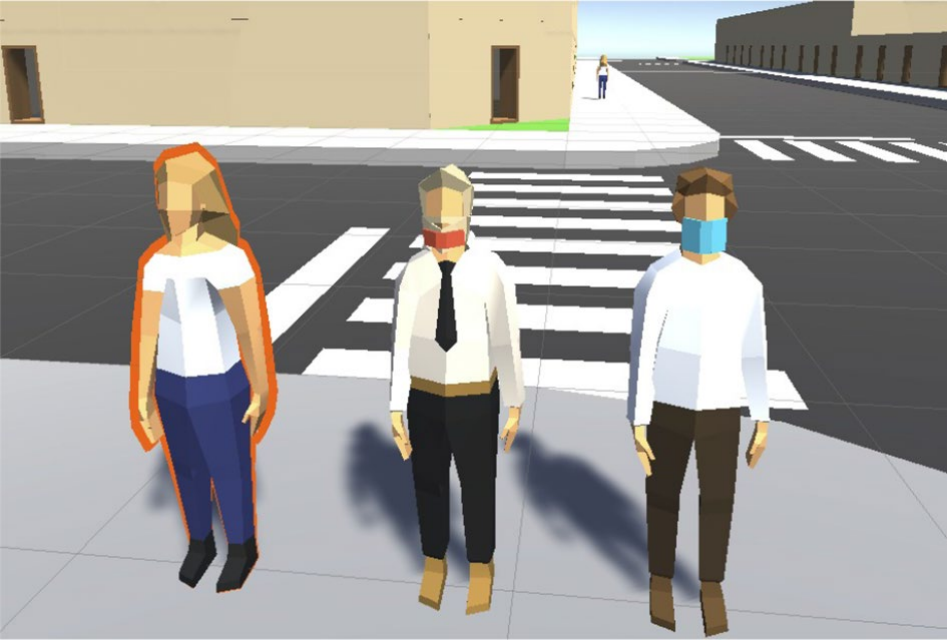
Illustration of agent not wearing a mask and infected (left), agent wearing it under the chin (middle), and agent wearing mask correctly.

Our simulation includes parameters that are important for the epidemiological study, such as the use of masks and the identification of infected agents. Visual identification of these epidemiological parameters may be important when making a detailed study of a particular situation. Thus, two types of masks can be observed on the agents: the first is a blue mask (Fig. 2, agent of the right), which indicates that the mask is well-fitted and offers a high range of protection; this mask covers the nose and mouth of the agent’s figure; the second is a red mask, placed under the chin (Fig. 2, agent in the middle), which symbolizes a poorly fitted mask. Additionally, an infected agent can be identified because its figure will be highlighted by a yellow to red border (Fig. 2, agent of the left), which indicates an infection and the viral load of the infection.

### Performance

In game engines such as Unity, performance is framerate-locked, meaning that if processor computational requirements are lower than the current availability, then the engine simply "waits" until the next frame to maintain a sustained framerate. However, our simulations depend on the number of agents used (i.e., each agent needs to be simulated), and thus they can overpass this limit, rendering simulations slower than the prescribed framerate and producing a visual lag between frames. This is a problem that cannot be avoided, as we are putting more agents in the simulation than what real-time requirement allows, resulting in a non-real-time simulation. This is not a problem for epidemiological studies but can have a negative impact on outreach purposes. In these cases, we recommend recording the simulation and visualizing it as a video.

On the other extreme, framerate-locking can be disabled, thus allowing faster-than-real-time simulations for a more moderate number of agents. All the examples in this manuscript did not reach the locking limitation, thus we were not forced to make any change in the simulation environment (i.e., Unity) settings for visualization purposes. However, 1-to-1 simulations are not desirable when drawing conclusions, e.g., for restriction measures, so we allowed our system to run several simulation steps for each rendered frame, resulting in a much faster simulation, allowing us to perform the whole simulation in a few minutes.

Finally, it must be noted that the simulation records all interactions and contacts between agents, so they can be used to compute disease spreading in different conditions. In our implementation, this has been implemented as a separate library, which runs almost instantaneously for all studied cases. However, it must be noted that this stage still depends on the number of interactions, which in turn depends on the number of agents in the first stage, so there is a dependency on this number, although much lighter in computational requirements.

## Results and discussion

### Case studies

**Figure 3:**
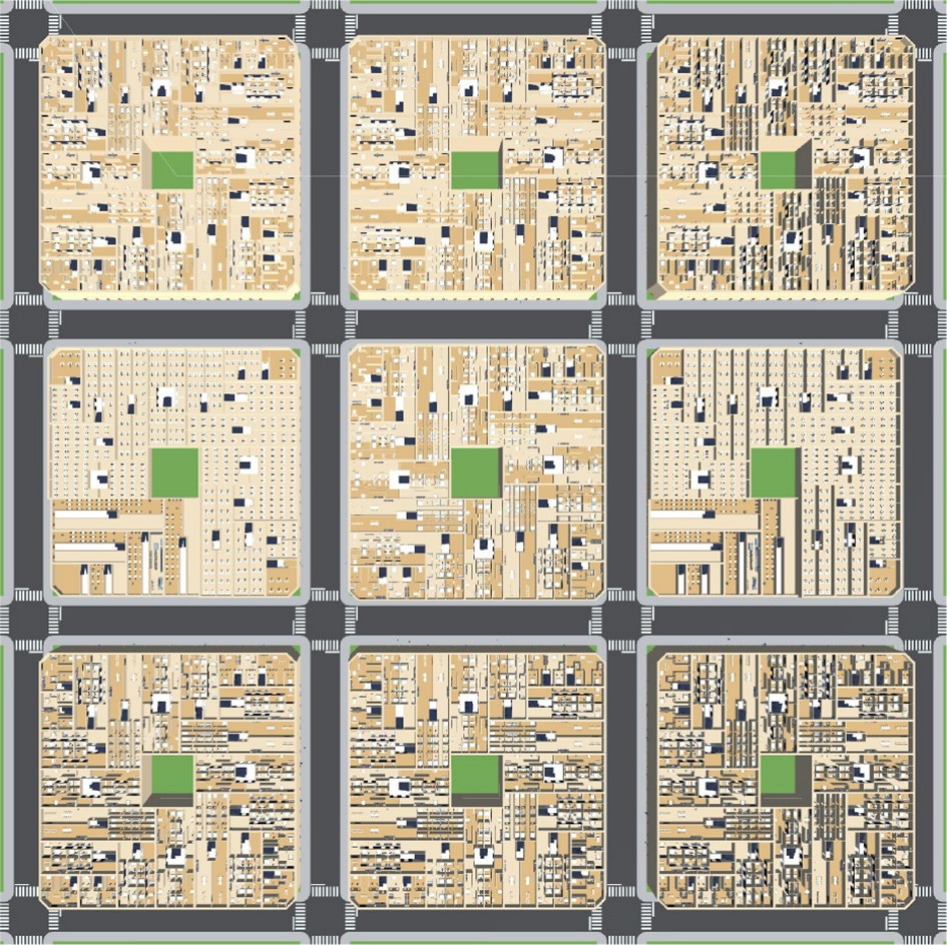
Illustration of a city created to run the experiments.

**Figure 4:**
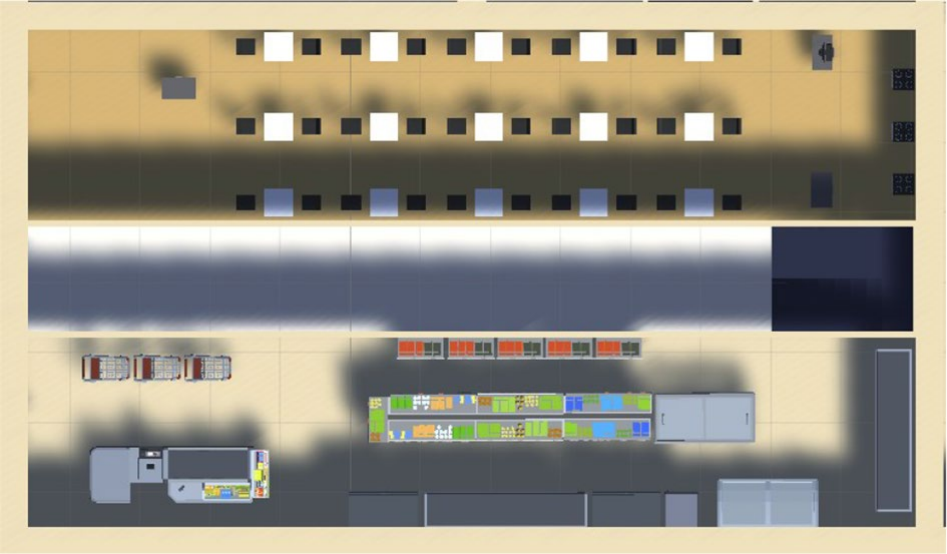
Top view of a restaurant (top) and a supermarket (bottom).

**Figure 5:**
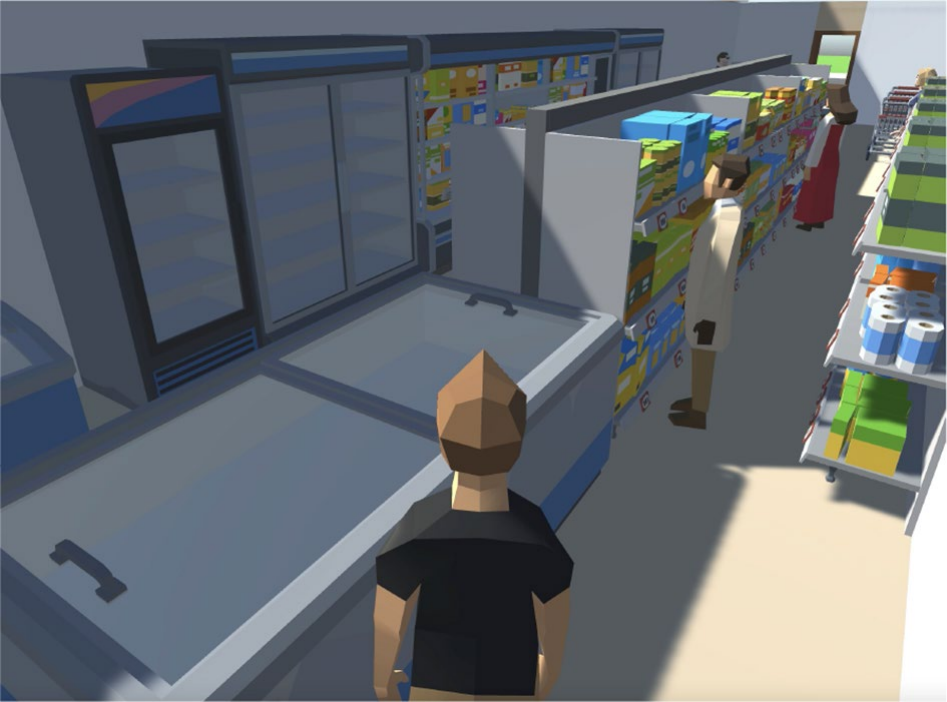
View inside a supermarket with agents interacting with the shelves.

For the experiment, we built a little city composed of 9 buildings that contain apartments, restaurants, supermarkets, and offices; there are 5376 agents living and interacting with the elements of the city. Figure 3 illustrates the map built to run the experiments. The three buildings at the top, the three buildings at the bottom, and the central building are 4-story residential buildings, with restaurants and supermarkets on floor 0. The buildings to the right and left of the central row are offices. Figure 4 shows a top view of a restaurant layout (top) and a supermarket layout (bottom). Figure 5 illustrates the view and interactions inside a supermarket; in our simulation, the agents arrive and interact with the supermarket, then it will retrieve five random shelves for the agent interactions, and finally, the agent will arrive to the paying queue in the cash register (these algorithms were copied from the restaurant’s welcome desk and cash register).

We prepared three scenarios where we varied the agendas followed by the agents. Each scenario represents a measure, ranging from reduced opening hours of night services to remote working and total confinement. On the one hand, we are going to compare the spread of the virus varying parameters like the type of mask used in the same scenario. On the other hand, we are going to make comparisons between the different scenarios that follow agendas that restrict the available activities like going to dinner in restaurants. The objective is to identify whether the measures taken against COVID-19 are conducive to stopping the spread of the virus. It is important to clarify that this model can be easily adapted to study any contagious virus, making variations in the infection parameters.

- Unrestricted scenario: The agendas in this scenario are complete with activities outside the houses, we have 4 types of agendas, one for people who work in offices, one for restaurant workers, another one for those who work in supermarkets, and, finally, one type for retired people or unemployed. The workers must follow a schedule and they have the option of having lunch and/or dinner in the available restaurants. Non-supermarket workers have a possible visit to the supermarket in the afternoon after work. Retired people or unemployed can go to restaurants and supermarkets during the day.
- Slightly restricted scenario: The agendas in this scenario are a bit restricted. They are very similar to the ones described in the previous scenario but with restrictions on the activities performed at noon, like going to restaurants or supermarkets at noon.
- Severely restricted scenario: The agendas in this scenario are very restricted, the agents in this simulation are working from home, and restaurants are not open. The only activity that can be performed by the agents is going to the supermarket.

### Results

Nine simulations were run corresponding to the three scenarios described in the previous section combined with three types of mask usage. For the study of mask usage, we first run a simulation with all the agents using masks of 75% to 98% protection. Secondly, the simulation was run with 10% of agents without masks, 25% of agents with masks worn incorrectly, and 65% of agents wearing masks as in the previous scenario; this simulation was called the "real scenario", due to its similarity to the times when the use of masks was mandatory throughout Europe but not all the people will respect the rules. Finally, a third simulation was run in which no agents wore a mask. All simulations had a duration of 5 days and the study with these three types of variations was performed for the three scenarios described in the previous section. Moreover, all scenarios and simulations were executed ten times, taking as a result of the analysis an average of the executions.

**Figure 6:**
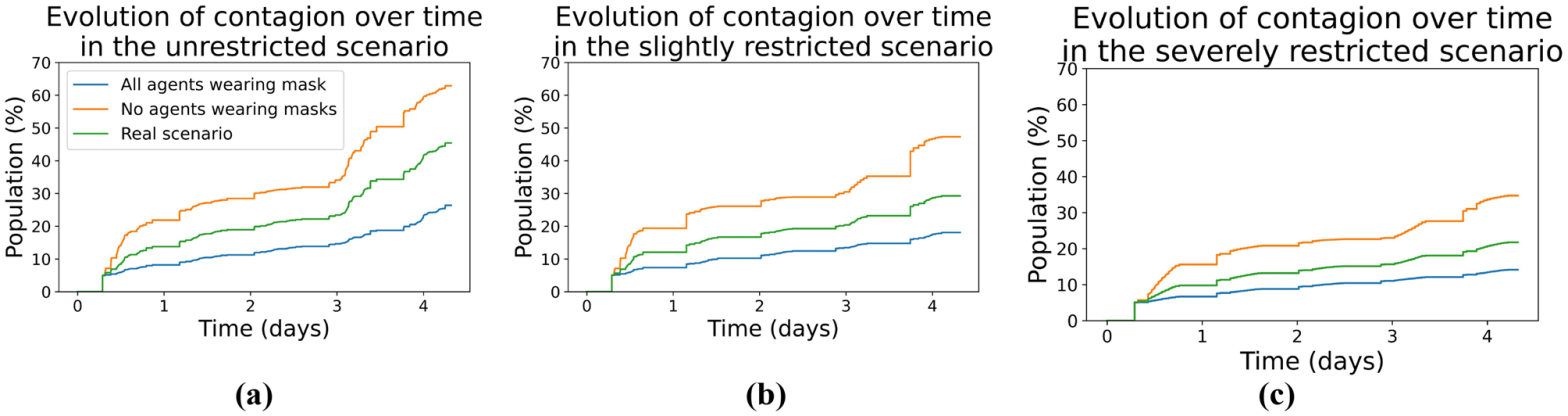
Comparison of contagion evolution over time when varying the type of mask usage: (**a**) unrestricted scenario, (**b**) slightly restricted scenario, and (**c**) severely restricted scenario.

Figure 6a shows for the unrestricted scenario a graph over time whose y-axis represents the percentage of infected agents, and the x-axis the timeline for 5 days of execution. It can be seen that the line representing the unmasked agent simulation (in orange) deviates considerably from the other two samples, indicating a higher percentage of infections. The line representing the real scenario (in green) is lower than the previous one, indicating a considerably lower number of infections; then, the line representing the simulation with all agents using a mask (in blue) shows the lowest number of infections for all three scenarios. If we observe closely this unrestricted scenario, we can see that at the end of the fifth day of the simulation, when no agents are wearing masks, we get 63% of the agents infected, while in the simulation of the real scenario, we get 45% of the agents infected and, in the simulation where all the agents wear masks, only 26% of the agents get infected, which represents almost a 40% difference with respect to the case with most agents infected. Additionally, we can observe the impact of the agents that were in the incubation period and start to be contagious from the third day onward. The graph shows the change in the slope of the cases of contagion after the third day, indicating a higher rate of contagion over time.

Figure 6b and c show for slightly restricted and severely restricted scenarios a graph over time where the y-axis represents the percentage of infected, and the x-axis the 5 days of execution. The behavior in both situations is very similar to that of the figure described above, the line corresponding to the simulation where the agents were not wearing masks is considerably higher than the other two, representing a higher rate of contagion. The lines representing the simulation with masks and the real scenario behave in a similar way, being slightly above the line of the real scenario again since there is a portion of unprotected agents in it.

All three scenarios show that the line representing the unmasked simulation is far higher than the other two lines. Further- more, it also shows that the slope of the graph on the third day is much steeper for the unmasked simulations. This demonstrates that the use of masks significantly influences the control of virus propagation. If we observe the lines across the three scenarios with increasing mobility restrictions, we can also observe the percentage of infected people decreases as restrictions increase. After making comparisons between the different types of variations within the same scenario, we will now compare the same variation between the different scenarios.

**Figure 7:**
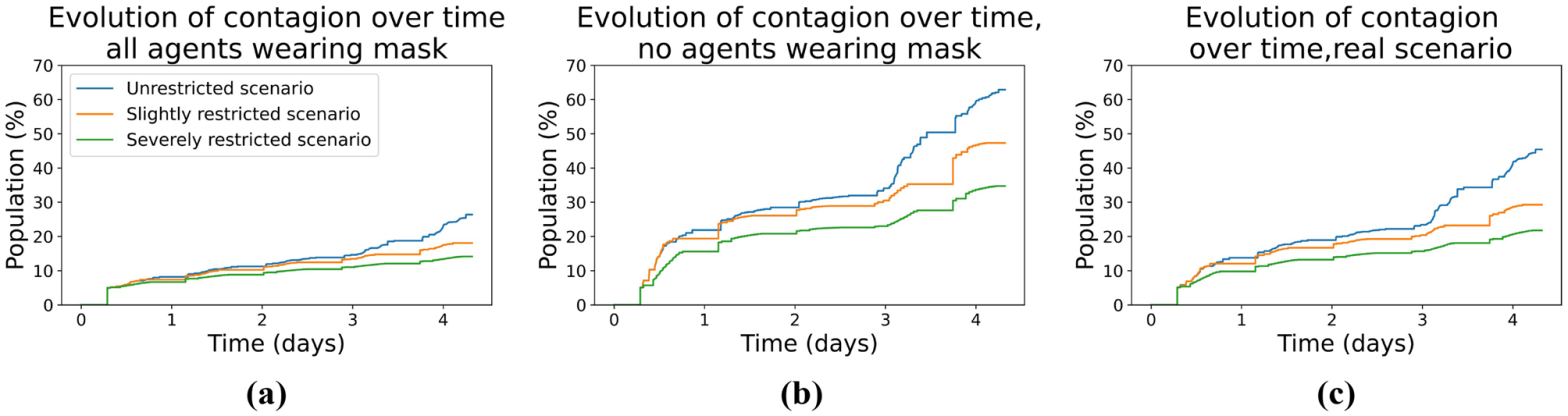
Comparison of contagion evolution over time for the three scenarios tested: (**a**) all the agents wearing a mask, (**b**) no agents wearing a mask, and (**c**) agents wearing masks as described in the real scenario.

First, we will study how the percentage of infected people behaves in the three scenarios with all agents using masks with 75% to 98% protection. Figure 7a shows how the infection percentages of the unrestricted scenario and the slightly restricted scenario are very similar, except on the third day when the infection line of the scenario with full activities begins to separate after the new infections go through the incubation period and are able to infect others. In the severely restricted scenario, where activities are limited to remote working and visits to the supermarket, the line remains below the other two, indicating the effectiveness of this scenario against the spread of the virus. At the end of the fifth day, in the unrestricted scenario, there are 26% infected, in the slightly restricted scenario there are 18% infected, and in the severely restricted scenario, there are 14% infected. The difference between the scenario with the most infected and the scenario with the least infected is almost double, which demonstrates the effectiveness of having restrictions. However, the small difference between slightly restricted and severely restricted could indicate that as long as masks are used correctly, it may not be necessary to impose severe mobility restrictions.

Secondly, we did the same experiment having no agents wearing masks. From Fig. 7b can be observed that the general contagion behavior of the three curves is similar. We can also observe that the percentage of the population infected in the severely restricted scenario is lower than in the other two scenarios, with both the curves being very close. If we compare the unrestricted scenario with 63% infected to the severely restricted scenario with 35% infected, the difference is almost twice as large. Finally, for the real scenario, we can observe in the curves from Fig. 7c that they have almost the same trajectory as the case described above, until the fourth day where the unrestricted scenario shows a higher percentage of infected.

**Figure 8:**
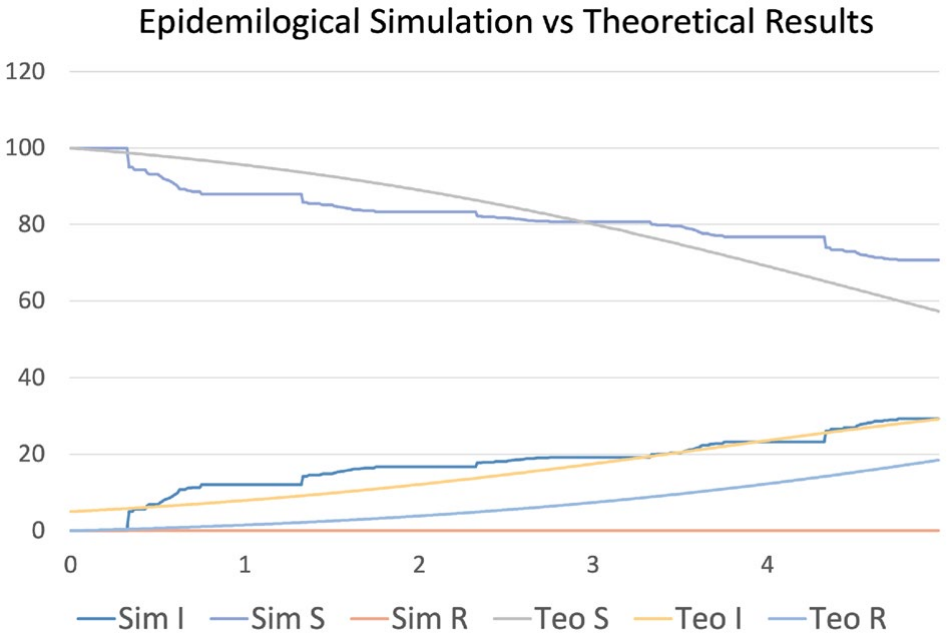
Comparison of the epidemiological simulation vs theoretical results. With I being infected, S being susceptible, and R being recovered.

Finally, Fig. 8 shows a comparison, for the slightly restricted scenario shown in Fig. 6b and the agents wearing masks as described for the "real scenario" case, with the theoretical SIR simulation model^15^ (SIR for Susceptible, Infected, Recovered). The three differential equations were integrated using a simple Euler optimization method, with a time step of ∆*t* = 0*.*25 days. The parameters used were fitted to the simulated data by a rough parameter sweep optimization, obtaining the values *β* = 0*.*03 and *γ* = 0*.*01, matching very closely the results from the literature for the recent COVID-19 pandemic^1^ and demonstrating the accuracy of the simulations involved. This not only demonstrates the capability of our micro-simulation framework to accurately represent fine-grained interactions but also shows its match with current state-of-the-art statistical population simulations.

On the other hand, our results for the different scenarios match quite closely the ones in the scientific literature using variants of the SIR model including/excluding masks and other important factors in virus spread^2,4,5,47^. In particular, if we compare the curves in Figs. 6 and 7 with the ones in the mentioned literature, we can observe an important match in the corresponding outcomes, again demonstrating the feasibility of the micro-simulation-based approach presented here with respect to results obtained at a global scale, and with the further advantage of allowing detailed contact tracing of individuals interactions.

Table 1 presents a slice of the full output of one such simulation. As we can see, at each time point, the system records all agent-agent or agent-place interactions. Possible interactions were reduced to four types: *ENTERCOLLIDER*_*PLACE*, *ENTERCOLLIDER*_*AGENT* , *EXITCOLLIDER*_*PLACE*, *EXITCOLLIDER*_*AGENT* , which allow recognizing entering/exiting spaces, or the start and end of a physical interaction between two agents. When an interaction of an agent with a place is recorded, all the agents inside that place at the moment of the new interaction are also recorded. Of course, using our framework, finer contacts within a building can also be traced by employing the same tools we have described, thus achieving an even greater level of detail. Table 2, in the Appendix, shows the detailed interaction of a single agent (i.e., agent ID 2243) throughout a normal simulation day. As all the building IDs correspond to modeled 3D buildings, we can reflect this information into a map showing all the interactions and their locations at the corresponding time-stamp. As we can see, this provides extremely detailed information on the whereabouts of a given agent at any moment.

**Table 1:** Example of the output of the simulation stage sent to the analysis stage.

Time	Subject ID	Actionname	Object ID
28985	4891	EXITCOLLIDER_PLACE	739411
28985	4355	EXITCOLLIDER_PLACE	260572
28985	4795	ENTERCOLLIDER_AGENT	4787
28985	4787	EXITCOLLIDER_PLACE	912006
28985	4787	EXITCOLLIDER_PLACE	118220
28985	4315	ENTERCOLLIDER_PLACE	532818

For each time point (first column), we record all interactions, which are simply recorded as the time stamp, the ID of the agent ("object") that generated an interaction, the type of interaction, and the ID of the object (agent or physical space) that the first agent interacted with. For instance, here we can see that agent 4891 went out of space 739411 at time point 28985 (first row).

**Table 2:** Example of contact tracing for agent 2243 for one of the simulations and for a given day.

Time	ID	Action	ID	Other agents
28860	2243	ENTERCOLLIDER_PLACE	442450	[]
28860	2243	ENTERCOLLIDER_PLACE	759649	[‘1283’, ‘1282’, ‘1281’, ‘1280’]
31549	2243	ENTERCOLLIDER_AGENT	2255	
31549	2243	ENTERCOLLIDER_AGENT	2255	
31549	2243	ENTERCOLLIDER_AGENT	2255	
31549	2243	ENTERCOLLIDER_AGENT	2255	
31550	2243	EXITCOLLIDER_AGENT	2255	
31550	2243	EXITCOLLIDER_AGENT	2255	
31550	2243	EXITCOLLIDER_AGENT	2255	
31550	2243	EXITCOLLIDER_AGENT	2255	
31605	2243	ENTERCOLLIDER_AGENT	1962	
31606	2243	ENTERCOLLIDER_AGENT	1962	
31606	2243	ENTERCOLLIDER_AGENT	1962	
31606	2243	ENTERCOLLIDER_AGENT	1962	
31606	2243	EXITCOLLIDER_AGENT	1962	
31606	2243	EXITCOLLIDER_AGENT	1962	
31606	2243	EXITCOLLIDER_AGENT	1962	
31607	2243	EXITCOLLIDER_AGENT	1962	
31672	2243	ENTERCOLLIDER_AGENT	1960	
31673	2243	EXITCOLLIDER_AGENT	1960	
31742	2243	ENTERCOLLIDER_AGENT	2248	
31743	2243	EXITCOLLIDER_AGENT	2248	
31785	2243	EXITCOLLIDER_PLACE	442450	[‘2243’, ‘83’]
31785	2243	EXITCOLLIDER_PLACE	759649	[‘1283’, ‘1282’, ‘1281’, ‘1280’, ‘2243’]
31861	2243	ENTERCOLLIDER_AGENT	1888	
31861	2243	ENTERCOLLIDER_AGENT	1888	
31861	2243	ENTERCOLLIDER_AGENT	1888	
31861	2243	ENTERCOLLIDER_AGENT	1888	
31861	2243	EXITCOLLIDER_AGENT	1888	
31861	2243	EXITCOLLIDER_AGENT	1888	
31861	2243	EXITCOLLIDER_AGENT	1888	
31862	2243	EXITCOLLIDER_AGENT	1888	
31948	2243	ENTERCOLLIDER_PLACE	60680	[‘2223’, ‘2230’, ‘2220’, ‘2222’, ‘2221’, ‘2228’, ‘2226’, ‘2227’, ‘2231’, ‘2224’]
31948	2243	ENTERCOLLIDER_PLACE	196950	[]
31948	2243	EXITCOLLIDER_PLACE	60680	[‘2223’, ‘2230’, ‘2220’, ‘2222’, ‘2221’, ‘2228’, ‘2226’, ‘2227’, ‘2231’, ‘2224’, ‘2243’]
31948	2243	EXITCOLLIDER_PLACE	196950	[‘2243’]
31953	2243	ENTERCOLLIDER_PLACE	655525	[‘2241’, ‘2235’, ‘2233’, ‘2239’, ‘2234’, ‘2240’, ‘2232’, ‘2242’, ‘2237’, ‘2236’]
50,247	2243	ENTERCOLLIDER_AGENT	2236	
50,248	2243	EXITCOLLIDER_AGENT	2236	
50,490	2243	EXITCOLLIDER_PLACE	655,525	[‘2235’, ‘2233’, ‘2239’, ‘2234’, ‘2232’, ‘2242’, ‘2237’, ‘2243’, ‘2238’]
50562	2243	ENTERCOLLIDER_AGENT	603	
50563	2243	ENTERCOLLIDER_AGENT	603	
50563	2243	ENTERCOLLIDER_AGENT	603	
50563	2243	ENTERCOLLIDER_AGENT	603	
50563	2243	EXITCOLLIDER_AGENT	603	
50563	2243	EXITCOLLIDER_AGENT	603	
50563	2243	EXITCOLLIDER_AGENT	603	
50564	2243	EXITCOLLIDER_AGENT	603	
52469	2243	ENTERCOLLIDER_PLACE	192612	[‘4231’, ‘4229’]
52470	2243	ENTERCOLLIDER_PLACE	192682	[‘4235’, ‘4233’, ‘4232’, ‘2178’, ‘2153’]
52471	2243	EXITCOLLIDER_PLACE	192612	[‘4231’, ‘4229’, ‘2243’]
57832	2243	ENTERCOLLIDER_PLACE	192612	[‘4231’, ‘4229’, ‘4228’]
57833	2243	EXITCOLLIDER_PLACE	192682	[‘4233’, ‘4232’, ‘2178’, ‘2153’, ‘2243’, ‘4256’]
57833	2243	EXITCOLLIDER_PLACE	192612	[‘4231’, ‘4229’, ‘4228’, ‘2243’]
57896	2243	ENTERCOLLIDER_AGENT	2583	
57897	2243	ENTERCOLLIDER_AGENT	2583	
57897	2243	ENTERCOLLIDER_AGENT	2583	
57897	2243	ENTERCOLLIDER_AGENT	2583	
57897	2243	EXITCOLLIDER_AGENT	2583	
57897	2243	EXITCOLLIDER_AGENT	2583	
57897	2243	EXITCOLLIDER_AGENT	2583	
57898	2243	EXITCOLLIDER_AGENT	2583	
58409	2243	ENTERCOLLIDER_AGENT	491	
58410	2243	EXITCOLLIDER_AGENT	491	
58472	2243	ENTERCOLLIDER_AGENT	1935	
58472	2243	ENTERCOLLIDER_AGENT	1935	
58472	2243	ENTERCOLLIDER_AGENT	1935	
58472	2243	ENTERCOLLIDER_AGENT	1935	
58472	2243	EXITCOLLIDER_AGENT	1935	
58472	2243	EXITCOLLIDER_AGENT	1935	
58472	2243	EXITCOLLIDER_AGENT	1935	
58472	2243	EXITCOLLIDER_AGENT	1935	
58473	2243	ENTERCOLLIDER_AGENT	3237	
58474	2243	EXITCOLLIDER_AGENT	3237	
58476	2243	ENTERCOLLIDER_AGENT	5187	
58477	2243	ENTERCOLLIDER_AGENT	5187	
58477	2243	EXITCOLLIDER_AGENT	5187	
58478	2243	EXITCOLLIDER_AGENT	5187	
58495	2243	ENTERCOLLIDER_AGENT	759	
58496	2243	ENTERCOLLIDER_AGENT	759	
58496	2243	ENTERCOLLIDER_AGENT	759	
58496	2243	ENTERCOLLIDER_AGENT	759	
58496	2243	EXITCOLLIDER_AGENT	759	
58496	2243	EXITCOLLIDER_AGENT	759	
58496	2243	EXITCOLLIDER_AGENT	759	
58497	2243	EXITCOLLIDER_AGENT	759	
58516	2243	ENTERCOLLIDER_PLACE	232966	[]
58516	2243	EXITCOLLIDER_PLACE	232966	[‘2243’]
58521	2243	ENTERCOLLIDER_PLACE	442450	[]
59010	2243	ENTERCOLLIDER_AGENT	2255	
59011	2243	EXITCOLLIDER_AGENT	2255	
71422	2243	EXITCOLLIDER_PLACE	442450	[‘2243’]
71445	2243	ENTERCOLLIDER_AGENT	759	
71446	2243	EXITCOLLIDER_AGENT	759	
71464	2243	ENTERCOLLIDER_AGENT	5187	
71465	2243	EXITCOLLIDER_AGENT	5187	
71511	2243	ENTERCOLLIDER_AGENT	2229	
71512	2243	EXITCOLLIDER_AGENT	2229	
71614	2243	ENTERCOLLIDER_AGENT	2937	
71615	2243	EXITCOLLIDER_AGENT	2937	
73612	2243	ENTERCOLLIDER_AGENT	5187	
73612	2243	ENTERCOLLIDER_AGENT	5187	
73613	2243	ENTERCOLLIDER_AGENT	5187	
73613	2243	ENTERCOLLIDER_AGENT	5187	
73613	2243	EXITCOLLIDER_AGENT	5187	
73613	2243	EXITCOLLIDER_AGENT	5187	
73613	2243	EXITCOLLIDER_AGENT	5187	
73613	2243	EXITCOLLIDER_AGENT	5187	
73631	2243	ENTERCOLLIDER_AGENT	759	
73631	2243	ENTERCOLLIDER_AGENT	759	
73631	2243	ENTERCOLLIDER_AGENT	759	
73631	2243	ENTERCOLLIDER_AGENT	759	
73632	2243	EXITCOLLIDER_AGENT	759	
73632	2243	EXITCOLLIDER_AGENT	759	
73632	2243	EXITCOLLIDER_AGENT	759	
73632	2243	EXITCOLLIDER_AGENT	759	
73650	2243	ENTERCOLLIDER_PLACE	232966	[‘2251’, ‘2874’]
73651	2243	EXITCOLLIDER_PLACE	232966	[‘2251’, ‘2874’, ‘2243’]
73655	2243	ENTERCOLLIDER_PLACE	442450	[]

The last column indicates the list of agents in the building at the moment of the entering/exiting events. The left column is the time elapsed since the beginning of the simulation.

From our experiment, it can be concluded that there is not much difference in contagion between an unrestricted scenario and a scenario where dining out and night shopping are not allowed, therefore, these kinds of measures may not be as needed to stop the spread. Moreover, the severely restricted scenario curves generally remain below the other two, indicating a lower percentage of infected agents and, thus it appears to be a useful measure to contain the spread of the virus and thus obtain better results. Furthermore, considering the results obtained, the number of infected persons almost tripled in all scenarios when comparing wearing masks against no masks, which is to be expected as our framework was written considering that wearing a mask reduces the probability of contagion^46^. This allows us to conclude that the measure that most influenced lowering the contagion rate was enforcing the use of masks with a high level of protection for the entire population.

## Conclusion

In this paper, we have presented a framework for the detailed simulation of agent-agent and agent-environment interactions in virtual cities with common components such as restaurants, supermarkets, and offices. Each agent has its own weekly agenda (different for working days and weekends) based on the semantics of the city, which describes the places it should interact with and the times when it should perform different actions. In addition, the semantic information stored within the objects of each location provides specific information for the agents about how they should behave inside those places. Finally, it is worth mentioning the close match between our simulations and the results obtained with the standard SIR theoretical model in the current epidemiology literature, demonstrating that the detailed simulations we propose scale adequately for a whole population simulation. However, we must emphasize that our framework is intended for tracing interactions up to the individual level, not to perform a whole-population statistical simulation.

At this point, we would like to clarify that, in our framework, Unity is merely a sophisticated tool that provides state-of-the- art collision detection and event handling, which are commonplace in video games, and which simplify the implementation burden considerably. On the other hand, it is also important to point out that our framework does not *compete*, but rather *complements* other developments such as GAMA, Emulsion, NetLogo, and Repast, as they could easily be extended to incorporate mechanisms for agent-agent or agent-environment interactions such as the ones described here. We aim to improve the state-of-the-art in virus-spread systems with these detailed interactions, which would allow an unprecedented level of contact tracing control at the simulations, gaining further insights into the contagion mechanisms.

A particularity of our tool is that it allows us to identify and register the contacts between agents, as well as their entrances and exits from closed spaces. This information can be used to model the propagation of any contagious virus whose behavior is based on these parameters, especially since we did experiments with parameters corresponding to COVID-19. We ran the system for three scenarios where we started with unrestricted schedules, adding restrictions on opening hours and teleworking; in addition, we considered the correct and incorrect use of the mask to study the impact of both restrictions and correct use of the mask on the spread of the virus. This propagation and simulation model can be useful to identify measures that may or may not be correct and accurate in the fight to reduce the spread of the virus. Again, observe the role of Unity as a practical geometry processing and organizing tool, allowing us to integrate the whole system in a single application. However, nothing prevents users from using other tools with similar geometry-creation/administration capabilities, such as Esri’s CityEngine^48^, at the added cost of forcing constant application switching, with the consequent problem of communicating the applications through files, which is cumbersome and error-prone.

The largest benefit of our framework, besides closely simulating the spread of the virus based on mask usage and mobility restrictions, is that we can track individuals and show information that is not available with other models. For example, we can follow an agent through the day and study the most likely sites where it can get infected. We can also extend the elements in the city by extending the current models and semantics to simulate in detail other sites of the city where agents interact. For example, we could incorporate a school where only kids interact during the day, and the parents meet briefly during pick up/drop off times, together with contagion properties in kids, and study the exact impact of opening schools. We believe that our microscopic agent simulation together with the visualization of the agents’ whereabouts, could become a powerful tool for policymakers to demonstrate the importance of their decisions.

## Data Availability

Code for this project will be provided by the corresponding author upon reasonable request.
